# EGFR 19和21外显子突变肺癌患者的临床特征比较和预后分析

**DOI:** 10.3779/j.issn.1009-3419.2014.11.06

**Published:** 2014-11-20

**Authors:** 仁旺 刘, 京豪 刘, 昕 李, 颖 李, 青春 赵, 作生 李, 红雨 刘, 军 陈

**Affiliations:** 1 300052 天津，天津医科大学总医院肺部肿瘤外科 Department of Lung Cancer Surgery, Tianjin Medical University General Hospital, 300052 Tianjin, China; 2 300052 天津，天津医科大学总医院，天津市肺癌研究所，天津市肺癌转移与肿瘤微环境重点实验 Tianjin Key Laboratory of Lung Cancer Metastasis and Tumor Microenvironment, Tianjin Lung Cancer Institute, Tianjin Medical University General Hospital, 300052 Tianjin, China

**Keywords:** 肺肿瘤, EGFR, 靶向治疗, 预后, Lung neoplasms, EGFR, Target therapy, Prognosis

## Abstract

**背景与目的:**

近年来，通过对表皮生长因子受体（epidermal growth factor receptor, EGFR）等相关信号通路的研究及对EGFR酪氨酸激酶抑制剂（EGFR-tyrosine kinase inhibitors, EGFR-TKIs）疗效及安全性的探索，证实了靶向治疗已成为肺癌的重要治疗手段之一。然而，在各中心试验中EGFR-TKIs应用于不同*EGFR*突变亚型患者的效果不尽相同，其原因尚有争议。因此，本研究通过对比EGFR 19和21外显子突变肺癌患者的临床特点和预后分析，以明确不同*EGFR*突变亚型肺癌患者的临床病理生理特征的差异，并为TKIs应用于EGFR不同突变亚型疗效差异的研究提供理论依据。

**方法:**

研究对象为EGFR外显子19或21突变阳性肺癌患者共113例。其中47例患者采用Real-time PCR或测序分析*EGFR*突变状况，余66例患者则是采用x-TAG液相芯片技术进行分析。收集临床资料，同时定期随访，以死亡作为患者终点事件，并应用SPSS 19.0软件进行统计学分析。

**结果:**

在本研究中，*EGFR*外显子突变阳性患者共113例。其中，19外显子突变患者56例，平均年龄为（57.02±11.31）岁；21外显子突变患者57例，平均年龄为（62.25±7.76）岁，两组患者在年龄分布上存在明显差异（*P*＜0.05）；EGFR外显子突变阳性患者以女性（61.9%）、非吸烟（72.6%）、腺癌（84.1%）多见，但两组患者在性别、吸烟状况、组织类型、分化程度及TNM分期等临床特征方面均无明显差异（*P*＞0.05）。进一步分析发现，19外显子突变患者相对21外显子突变患者多好发于右侧（*P*＜0.05）。对所有具有完整随访资料的91例（80.53%, 91/113）患者预后分析发现，两组患者总生存期无统计学差异（中位生存期19外显子组和21外显子组分别为1, 051 *vs* 1, 076天，*P*=0.566）。进一步分析发现，在年龄＞61岁、男性、吸烟、Ⅳ期肺癌患者中，19外显子突变组患者均表现出相对较好的预后，但均无统计学差异。

**结论:**

*EGFR* 19外显子突变肺癌患者相对21外显子突变肺癌患者年龄较小，且右侧原发较为多见。两者在性别、吸烟状况、组织类型、分期及分化程度上无明显差别。两者总生存时间无差异，但仍然需要进一步研究论证。

## Abstract (en)

**Background and objective:**

Studies on the epidermal growth factor receptor (EGFR) signaling pathways and the therapeutic effects of EGFR-tyrosine kinase inhibitors (EGFR-TKIs) have recently proven that targeted therapy has a major role in the treatment of lung cancer. However, the therapeutic effects of EGFR-TKIs on lung cancers with different *EGFR* mutation subtypes remain unclear. And if there is a significant difference in the effects of EGFR-TKIs, the mechanisms for the difference remain unclear. The aim of this study was to investigate the clinical importance of *EGFR* mutations in exons 19 and 21 of lung cancer patients and to compare the outcomes of these patients.

**Methods:**

The study recruited 113 patients who had non-small cell lung cancer (NSCLC) with *EGFR* mutations. *EGFR* mutations were detected for 47 patients using Real-time PCR or DNA sequencinag. The mutations of the remaining patients were determined using xTag-EGFR liquid chip technology. All stages Ⅰ-Ⅲ patients underwent radical resection followed by 4 cycles of postoperative chemotherapy. Patients with pleural metastases underwent pleural biopsy, pleurodesis, and chemotherapy only. Patients with distant metastases underwent biopsy and chemotherapy only. Collected clinical data were analyzed using SPSS 19.0 software.

**Results:**

*EGFR* exon mutations 19 and 21 were found in 56 and 57 patients, respectively. The mean age of patients with exon 19 mutations was lower than the age of the patients with exon 21 mutations (57.02±11.31 years *vs* 62.25±7.76 years, respectively; *P* < 0.05). The primary tumors of patients with exon 19 mutations were more likely occur in the right lung. There were no significant differences in gender, smoking status, histopathology, level of differentiation, and stage of disease (*P* > 0.05) between the patients with exon 19 and 21 mutations; and survival analysis of 91 (80.5%) patients with complete clinical data found no differences in overall survival. Stratification analysis found out that patients with exon 19 mutations had longer overall survival associated with age > 61 years, male gender, ever smoking, and stage Ⅳ disease; although the differences were not significant.

**Conclusion:**

Compared to the lung cancer patients with *EGFR* exon 21 mutations, the patients with *EGFR* exon 19 mutations were younger, and their primary tumors were more likely to occur in the right lung. There were no significant differences between the lung cancer patients with exon 19 and 21 mutations for overall survival, gender, smoking status, histopathology, level of differentiation, and disease stage.

肺癌已经成为全球范围内肿瘤患者致死的最主要病因之一，其发病率和死亡率仍然居高不下^[1]^。传统的治疗方式包括外科手术治疗，化疗及放疗等，在取得一定疗效的同时，由于较大的副作用而降低了患者的生活质量。近年来，通过对表皮生长因子受体（epidermal growth factor receptor, EGFR）等相关信号通路的研究及对EGFR酪氨酸激酶抑制剂（EGFR-tyrosine kinase inhibitors, EGFR-TKIs）疗效及安全性的探索，证实了EGFR-TKIs在*EGFR*突变的肺癌患者中取得确切的疗效，同时副反应轻，从而改善了患者的生活质量^[2, 3]^。因此，靶向治疗因其给药方式简便、高特异性、耐受性好、无严重副作用等原因，已成为肺癌的重要治疗手段之一。

EGFR是上皮源性恶性肿瘤的最重要的一个分子之一，其活性与肿瘤的生长、侵袭和转移密切相关^[4]^。在肺癌中，EGFR-TKIs治疗的优势人群为亚裔、女性、非吸烟的患者，因为其多具有EGFR外显子的突变。EGFR的主要突变位点包括18-21外显子，其中以19外显子的缺失突变和21外显子的点突变最为常见^[5]^。然而最近研究表明，在各中心试验中EGFR-TKIs应用于不同*EGFR*突变类型患者的效果不尽相同。Lee等^[6]^通过对比170例*EGFR*突变阳性非小细胞肺癌（non-small cell lung cancer, NSCLC）患者一线应用TKIs疗效及预后发现，不同亚型的*EGFR*突变患者应用EGFR-TKIs疗效不同。其中，E746起始的19外显子缺失突变患者应用TKIs药物后相对L747起始的缺失突变患者无进展生存期（progression free survival, PFS）更长；同时，21外显子L858R突变患者PFS比L861R/L861Q更长。李俭杰等^[7]^亦有报道，通过比较19外显子缺失突变及21外显子点突变晚期NSCLC患者一线应用TKIs类药物疗效，发现外显子19缺失患者获益更明显，表现出有较好的PFS和总生存期（overall survival, OS）。

不同亚型的*EGFR*突变患者应用TKIs药物时疗效不同的原因尚有争议，可能与TKIs药物在不同亚型突变患者中动力学差异相关^[8]^，也可能与肿瘤自身特性相关。因此，本研究以天津医科大学总医院肺部肿瘤外科收治的113例EGFR阳性患者为研究对象，对比EGFR 19外显子缺失突变和21外显子点突变患者的临床特点，同时进行预后分析，目的是为了明确EGFR不同突变亚型的临床特点及预后差异，旨在为后期对TKIs药物应用于EGFR不同突变亚型的疗效差异原因的研究提供理论依据。

## 1 材料与方法

### 1.1 临床资料

选取2007年7月1日-2012年7月1日天津医科大学总医院肺部肿瘤外科收治的EGFR外显子19或21突变阳性肺癌患者共113例。其中，男性43例，女性70例；年龄33岁-78岁，中位年龄为61岁；吸烟患者31例，非吸烟82例；左侧原发49例，右侧原发64例；按世界卫生组织（World Health Organization, WHO）肺癌组织分类标准进行组织分型，其中鳞癌6例，腺癌95例，腺鳞癌7例，大细胞肺癌4例，肉瘤样癌1例；高分化57例，中分化37例，低分化12例，另有7例患者为活检确诊，未能确定分化程度；根据国际抗癌联盟（Union for International Cancer Control, UICC）1997年新的修订标准进行肿瘤-淋巴结-转移（tumor node metastasis, TNM）分期，Ⅰ期31例，Ⅱ期15例，Ⅲ期42例，Ⅳ期25例。

所有患者均通过手术病理或穿刺活检病理确诊，其中47例患者采用Real-time PCR或测序分析肺癌患者石蜡组织中EGFR外显子19、21突变状况，余66例患者则是采用x-TAG液相芯片技术进行分析。Ⅰ期-Ⅲ期患者均行肺癌根治术，术后给予4周期辅助化疗，其中鳞癌选用吉西他滨联合顺铂方案，腺癌选用培美曲塞联合顺铂治疗。对于Ⅳ期患者，仅胸膜转移者行开胸活检，种植结节灼烧术，胸膜固定术后给予化疗，方案同上；有远处转移患者共7例，行穿刺活检明确病理后，给予化疗，方案同上。所有标本均以10%福尔马林固定，常规石蜡包埋封存。生存时间从手术后或穿刺后第1天算起，截止至2014年7月25日。

### 1.2 Real-time PCR或sequence检测*EGFR*突变

采用Real-time PCR或sequence分析各样本中*EGFR*基因突变状况（包括delE746-A750、delL747-P753insS、L858R及L861Q），详见本中心已发表的文献^[9, 10]^。此两种方法共检测出*EGFR*突变47例。

### 1.3 xTAG液相芯片技术检测*EGFR*突变

采用xTAG-液相芯片技术检测，由广州益善公司完成。具体方法如下：①从样本中抽提DNA。②多重PCR扩增：将抽提出的DNA（包括突变型和野生型DNA）在同一PCR反应体系中同时扩增。③消化处理：通过酶处理第一轮PCR反应产物，去除多余的引物及游离的核苷酸，防止多余的引物及核苷酸与后续的探针非特异结合。④等位基因特异性延伸（allele-specific primer extension, ASPE）：为分辨不同的突变位点，每个目的基因序列都设计有相应的ASPE引物，引物的一端与目的基因序列特异性的结合，在此过程中，系统中掺入有生物素标记的脱氧核糖核苷三磷酸（deoxy-ribonucleoside triphosphate, dNTP），如果目的基因序列与探针序列相匹配，则可以继续延伸，并使反应终产物上连有多个生物素，若两者不匹配则延伸终止，反应终产物上没有生物素结合；在引物另一端连接着Tag标签序列，这些序列将与微球上特异的Anti-tag序列相结。⑤杂交反应：将扩增产物与交联了特异探针的微球进行杂交反应。⑥Luminex阅读仪上读取数据：将杂交后的产物放入Luminex仪器进行读数，根据微球自身发出荧光色判别球号以及对应的检测基因，根据微球发出的荧光强度判别是否有突变。此方法共检测出*EGFR*突变66例。

### 1.4 统计学方法

采用SPSS 19.0软件进行数据统计分析。应用*Mann*-*Whitney*检验对方差不齐的两组计量资料进行分析，应用χ^2^检验对计数资料进行分析，应用*Cox*回归模型分析预后因素，应用*Kaplan*-*Meier*曲线进行生存分析，以*P*＜0.05为差异具有统计学意义。

## 2 结果

### 2.1 EGFR 19、21外显子突变患者一般情况的对比

本研究中，入组患者共113例，其基本特征及临床特点详见表 1。其中，女性（61.9%）、非吸烟（72.6%）、腺癌（84.1%）多见，与文献报道一致。检测出EGFR 19外显子突变患者共56例，21外显子突变患者共57例。其中19外显子突变患者平均年龄（57.02±11.31）岁，21外显子突变患者平均年龄（62.25±7.76）岁，差异有统计学意义（*P*＜0.05）。两组患者均以女性（19外显子 *vs* 21外显子：66.1% *vs* 57.9%）、非吸烟（19外显子 *vs* 21外显子为73.2% *vs* 71.9%）多见，19、21外显子突变患者在不同性别和吸烟状况中的分布频率差异无统计学意义（*P*＞0.05）。两组患者基本特征详见表 2。

**1:** 入组患者的基本情况 Basic information of 113 patients with *EGFR* mutaion

Characteristic	Number	Percentage
Gender		
Male	43	38.1%
Female	70	61.9%
Smoke status		
Never	82	72.6%
Ever	31	27.4%
Primary site		
Left side	49	43.4%
Right side	64	56.6%
Histology		
Adenocarcinoma	95	84.1%
SCC	6	5.3%
Ad-SCC	7	6.2%
Large cell lung cancer	4	3.5%
Lung sarcomatoid carcinoma	1	0.9%
Differentiation^*^		
Well	57	50.4%
Moderately	37	32.7%
Poorly	12	10.6%
TNM staging		
Ⅰ	31	27.4%
Ⅱ	15	13.3%
Ⅲ	42	37.2%
Ⅳ	25	22.1%
^*^The differentiation of 7 patients in this study is not defined. SCC: squamous cell carcinoma.		

**2:** EGFR 19和21外显子突变患者临床特征的比较 Different clinical features between exon 19 and 21 mutations

Parameters		*EGFR* mutation		*Z*/*χ*^2^	*P*
		Exon 19 (*n*=56)	Exon 21 (*n*=57)		
Age (yr)		57.02±11.31	62.25±7.76	-2.451	0.014
Gender	Male	19 (33.9%)	24 (42.1%)	0.801	0.371
	Female	37 (66.1%)	33 (57.9%)		
Somke status	Never	41 (73.2%)	41 (71.9%)	0.023	0.878
	Ever	15 (26.8%)	16 (28.1%)		

### 2.2 EGFR 19、21外显子突变患者病理生理学特点分析

通过临床资料收集，进行统计学分析，对比出19、21外显子突变患者肿瘤特点的差异，详见表 3。在肿瘤原发位点方面，19相对21外显子突变患者多好发于右侧（19外显子 *vs* 21外显子为66.1% *vs* 47.4%, *P*=0.045）。两组患者均以腺癌（外显子19 *vs* 外显子21：80.4% *vs* 87.7%，*P*=0.738），高分化肿瘤（19外显子 *vs* 21外显子：57.1% *vs* 43.9%, *P*=0.621）多见，差异无统计学意义。另外，两组资料TNM分期无明显差异（Ⅰ期：19外显子 *vs* 21外显子：33.9% *vs* 21.1%；Ⅱ期：19外显子 *vs* 21外显子：10.7% *vs* 15.8%；Ⅲ期：19外显子 *vs* 21外显子：35.7% *vs* 38.6%；Ⅳ期：19外显子 *vs* 21外显子：19.6% *vs* 24.6%, *P*=0.453）。

**3:** EGFR 19和21外显子突变患者病理生理特征的比较 Comparison of clinical significance between EGFR exon 19 mutation groups and EGFR exon 21 mutation groups

Characteristic		*EGFR* mutation		*χ*^2^	*P*
		Exon 19 (*n*=56)	Exon 21 (*n*=57)		
Primary site	Left side	19 (33.9%)	30 (52.6%)	4.023	0.045
	Right side	37 (66.1%)	27(47.4%)		
Histology	Adenocarcinoma	45 (80.4%)	50 (87.7%)	1.264	0.738
	SCC	4 (7.1%)	2 (3.5%)		
	Ad-SCC	4 (7.1%)	3 (5.3%)		
	Others	3 (5.4%)	2 (3.5%)		
Differentiation^**^	Well	32 (57.1%)	25 (43.9%)	0.953	0.621
	Moderately	17 (30.4%)	20 (35.1%)		
	Poorly	6 (10.7%)	6 (10.5%)		
TNM staging	Ⅰ	19 (33.9%)	12 (21.1%)	2.627	0.453
	Ⅱ	6 (10.7%)	9 (15.8%)		
	Ⅲ	20 (35.7%)	22 (38.6%)		
	Ⅳ	11 (19.6%)	14 (24.6%)		
^**^The differentiation of one patient with EGFR exon 19 mutation and 6 patients with EGFR exon 21 mutation is not defined in this study.					

### 2.3 EGFR 19、21外显子突变患者预后分析

截止到2014年7月25日，在113例*EGFR*突变患者中，有22例患者失访，31例患者死亡。将患者分为19外显子突变组和21外显子突变组，以患者死亡为事件终点。19外显子突变组患者失访12例，中位生存期1, 051天，而21外显子突变组患者失访10例，中位生存期1, 076天，两者总生存期无统计学差异（*P*=0.566，图 1）。应用*Cox*回归分析比较各因素风险比发现，高、中分化患者风险较低（高分化 *vs* 低分化，HR=0.203，95%CI: 0.076-0.543，*P*=0.001；中分化 *vs* 低分化，HR=0.203，95%CI：0.069-0.594，*P*=0.004），其余各项因素均无统计学意义。进一步，我们对患者进行分化程度的分层分析，发现在高中低各分化组患者中，19、21外显子突变患者未表现出明显差异，且无统计学意义（图 2）。

**1:**
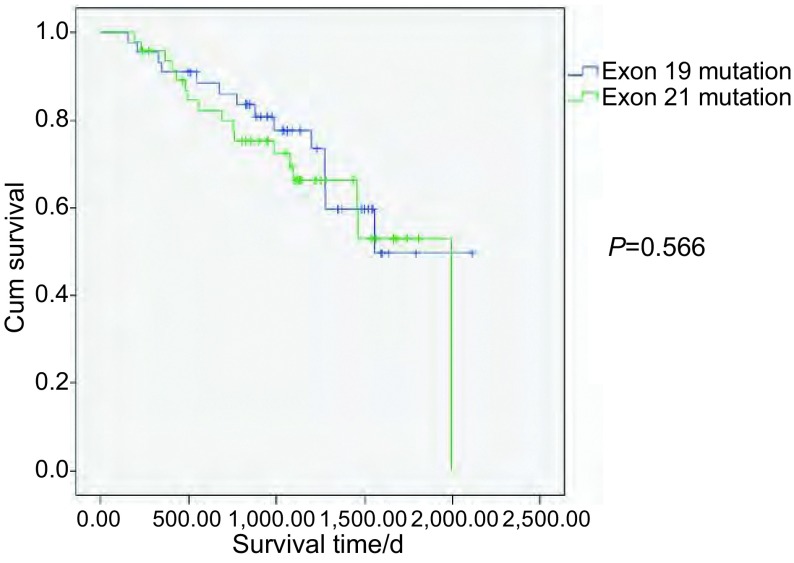
EGFR 19和21外显子突变患者总生存期的对比 The overall survival analysis of the patients with EGFR exon 19 & 21 mutations. EGFR: epidermal growth factor receptor.

**2:**
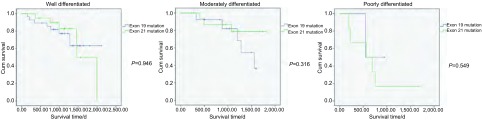
以分化程度分层分析对比EGFR 19和21外显子突变患者预后的差异 Comparison of survival curves between EGFR exon 19 mutation groups and exon 21 mutation groups, adjusted to differentiation.

随后，我们对两组患者分别就年龄、性别、吸烟情况对比分析19外显子突变患者和21外显子突变患者的预后情况。根据中位年龄，将病例分为≤61岁组和＞61岁组，发现≤61岁组中19、21外显子突变患者预后无明显差异；在＞61岁组中19外显子突变患者相对较好，但无统计学意义（图 3A、图 3B）。对性别进行分层分析发现，在女性患者中，19、21外显子突变患者预后无统计学差异；男性患者中，19外显子突变患者相对较好，但无统计学意义（图 3C、图 3D）。对吸烟状况进行分层分析发现，对于非吸烟患者，19外显子突变患者预后相对较差，但差异无统计学意义；对于吸烟患者，19外显子突变患者预后相对较好，但差异仍无统计学意义（图 3E、图 3F）。

**3:**
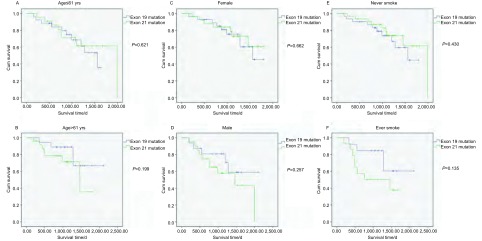
以年龄、性别、吸烟状态进行分层分析，对比EGFR 19和21外显子突变患者预后的差异 Comparison of survival curves between EGFR exon 19 mutation groups and exon 21 mutation groups, adjusted to age, gender and smoking status.

进一步，我们对两组患者分别根据分期进行分层分析。在各期中19、21外显子突变患者预后差异均无统计学意义，但对于Ⅰ期和Ⅱ期患者而言，19外显子突变患者预后表现出较差的趋势；Ⅲ期患者中两种突变类型的预后差异不明显；Ⅳ期患者中19外显子突变患者预后有较好趋势（图 4）。

**4:**
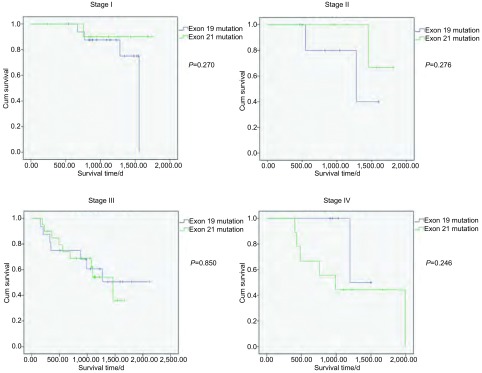
对比不同分期的EGFR 19和21外显子突变患者预后的差异 Comparison of survival curves between EGFR exon 19 mutation groups and exon 21 mutation groups, adjusted to clinical stage.

## 3 讨论

19、21外显子突变肺癌患者临床特点的差异尚有争议。有文献^[11]^报道，EGFR 21外显子突变肺癌患者中，以女性、肿瘤右侧原发、淋巴结转移阳性及Ⅲ期、Ⅳ期患者多见，但未分析比较两者预后差异。国内学者^[12]^亦有报道，19外显子突变肺癌患者相对21外显子突变肺癌患者而言女性突变率较高。与上述文献不同的是，本实验中剔除了*EGFR*未突变的患者，没有比较19及21外显子突变患者在所有肺癌患者中比例的差异，而是直接对比两组患者各参数的比例的差异，着重于19、21外显子突变患者在年龄、性别、吸烟状况、原发部位、组织类型、分期及分化程度的自然发生率的对比。我们中心研究发现，19外显子突变患者较21外显子突变患者年龄较小，且与文献报道不同的是19外显子突变患者右侧原发较为多见。两者在性别、吸烟状况、组织类型、分期及分化程度上无统计学差异。同样的方法，Riely等^[13]^通过对比美国人口中19、21外显子突变肺癌患者差异发现，两者在性别、种族、吸烟状况及组织类型的自然发生率上亦无差异。

我们研究中发现，在传统术后一线辅助化疗的治疗方案中，19、21外显子突变肺癌患者总生存时间无统计学差异。进而，我们对患者进行多因素回归分析，发现分化程度是影响患者预后的危险因素。因此，我们对患者进行分化程度的分层分析，发现两组患者并未表现出差异，表明EGFR 19、21外显子突变肺癌患者在传统治疗模式的基础上预后无明显差异，而由于没有完整的TKIs治疗的临床资料，故在本组病例中不能分析TKIs的疗效，因此，两组患者对EGFR-TKIs的疗效的差异需要进一步探讨。

我们进一步对各个因素分别进行分层分析发现，分别在高龄、男性、吸烟的*EGFR*突变肺癌患者中，在应用传统术后一线辅助化疗方案时，19外显子突变患者均有较好的预后趋势，但差异无统计学意义。在对分期进行分层分析，发现早期患者中，19外显子突变患者预后表现出较差的趋势，而Ⅳ期患者中19外显子突变患者预后有较好趋势，但仍无统计学意义。随访时间较短，病例数较少可能是两者预后差异有趋势但无统计学意义的主要原因。后续研究应扩大样本量，以明确两者之间趋势有无统计学意义。同时，细分EGFR 18-21外显子突变的不同亚型，对比各亚型的临床特点、传统治疗预后差异及TKIs药物应用后预后差异，旨在为论证不同*EGFR*突变亚型患者应用TKIs药物效果不同的原因提供理论依据，同时指导TKIs药物对不同*EGFR*突变亚型患者的临床应用。

关于两者预后的对比的报道尚不多见，Riely等^[13]^通过对比EGFR 19或21外显子突变共34例肺癌患者在应用TKIs药物的效果，发现19外显子突变患者PFS及OS明显优于21外显子突变患者。亦有文献^[14]^报道，EGFR缺失突变肺癌患者相比其他*EGFR*突变类型肺癌患者而言对吉非替尼的反应率更高。Zhu等^[15]^从细胞水平分析了EGFR19外显子缺失突变和21外显子点突变对吉非替尼反应性不同的原因，发现吉非替尼均能导致EGFR 19外显子缺失突变和EGFR 21外显子点突变的稳定转染细胞EGFR、Akt和Erk分子磷酸化受到抑制，但是，对前者的抑制较后者更明显。而与21外显子点突变的细胞比较，吉非替尼能导致更多的19外显子缺失的细胞G_1_期阻滞。如果从分子水平分析，19外显子的缺失突变和21外显子的点突变均发生于EGFR分子的酪氨酸激酶区，从空间构象上看，19外显子位于EGFR的αC-helix区域，而21外显子位于EGFR分子的A-loop区^[16]^。Sordella等^[17]^报道，19外显子突变和21外显子突变能导致EGFR分子自身磷酸化的位点不一样，而导致其下游的信号通路不同，比如，相对于缺失突变，L858R突变中的845密码子编码的酪氨酸残基表现出高度磷酸化的状态。这可能是19外显子突变肺癌患者相对21外显子突变肺癌患者对TKIs药物反应率较高，应用TKIs药物后预后较好的原因。

综上所述，EGFR 19外显子突变肺癌患者相对21外显子突变肺癌患者年龄较小，且右侧原发较为多见。两者在性别、吸烟状况、组织类型、分期及分化程度上无明显差别。EGFR 19、21外显子突变肺癌患者总生存时间无明显差异，但仍然需要进一步研究论证。
